# 4377 Missed Opportunities to Prevent Homicide: An Analysis of the National Violent Death Reporting System

**DOI:** 10.1017/cts.2020.413

**Published:** 2020-07-29

**Authors:** Justin Cirone, Jennifer Cone, Brian Williams, David Hampton, Priya Prakash, Tanya Zakrison

**Affiliations:** 1 University of Chicago

## Abstract

OBJECTIVES/GOALS: The goal of this study is to better understand the homicide victim population who were institutionalized within 30 days prior to their death. Improved knowledge of this population can potentially prevent these future homicides. METHODS/STUDY POPULATION: A retrospective analysis of the 36 states included in the 2003-2017 National Violent Death Reporting System was performed. Demographics of recently institutionalized homicide victims (RIHV) in the last 30 days were compared to homicide victims who were not recently institutionalized. Circumstances of the homicide, such as suspected gang involvement, were also compared. Parametric and non-parametric statistical analyses were performed. Significance was set at p<0.05. RESULTS/ANTICIPATED RESULTS: There were 81,229 homicides with 992 (1.2%) RIHV. The majority of RIHV were Black (49.6%) and older than victims who were not recently institutionalized (37.2 vs. 34.8, p<0.001). RIHV had a high school degree or higher in 54.8% of cases and the primary homicide weapon was a firearm in 67% of the deaths. They were more likely to be homeless (3.1% vs. 1.5%), have a mental health diagnosis (9.2% vs. 2.3%), abuse alcohol (6.1% vs. 2.2%), or abuse other substances (15.2% vs. 5.8%) [all p <0.001]. These victims were most commonly institutionalized in a correctional facility or a hospital compared to other facilities such as nursing homes. Homicide circumstances for RIHV were more likely to involve abuse/neglect (4.3% vs. 2.2%, p<0.001), gang violence (7.6% vs. 5.6%, p = 0.002), or a hate crime (1.0% vs. 0.1%. p<0.001). DISCUSSION/SIGNIFICANCE OF IMPACT: Contact with an institution such as a hospital or prison provides high-risk patients the opportunity to potentially participate in violence intervention programs. These institutions should seek to identify and intervene on this population to reduce the risk of violent homicides.

